# Surveillance for Malaria Elimination in Swaziland: A National Cross-Sectional Study Using Pooled PCR and Serology

**DOI:** 10.1371/journal.pone.0029550

**Published:** 2012-01-06

**Authors:** Michelle S. Hsiang, Jimee Hwang, Simon Kunene, Chris Drakeley, Deepika Kandula, Joseph Novotny, Justin Parizo, Trevor Jensen, Marcus Tong, Jordan Kemere, Sabelo Dlamini, Bruno Moonen, Evelina Angov, Sheetij Dutta, Christian Ockenhouse, Grant Dorsey, Bryan Greenhouse

**Affiliations:** 1 Global Health Group, University of California San Francisco, San Francisco, California, United States of America; 2 Department of Pediatrics, University of California San Francisco, San Francisco, California, United States of America; 3 Malaria Branch, Centers for Disease Control and Prevention, Atlanta, Georgia, United States of America; 4 Malaria Control Program, Department of Health, Manzini, Swaziland; 5 Department of Infectious and Tropical Diseases, London School of Hygiene and Tropical Medicine, London, United Kingdom; 6 Clinton Health Access Initiative, Boston, Massachusetts, United States of America; 7 School of Medicine, University of California San Francisco, San Francisco, California, United States of America; 8 School of Engineering and Applied Science, University of California Los Angeles, Los Angeles, California, United States of America; 9 University of North Carolina, Chapel Hill, North Carolina, United States of America; 10 Division of Malaria Vaccine Development, Walter Reed Army Institute of Research, Silver Spring, Maryland, United States of America; 11 Department of Medicine, University of California San Francisco, San Francisco, California, United States of America; Menzies School of Health Research, Australia

## Abstract

**Background:**

To guide malaria elimination efforts in Swaziland and other countries, accurate assessments of transmission are critical. Pooled-PCR has potential to efficiently improve sensitivity to detect infections; serology may clarify temporal and spatial trends in exposure.

**Methodology/Principal Findings:**

Using a stratified two-stage cluster, cross-sectional design, subjects were recruited from the malaria endemic region of Swaziland. Blood was collected for rapid diagnostic testing (RDT), pooled PCR, and ELISA detecting antibodies to *Plasmodium falciparum* surface antigens. Of 4330 participants tested, three were RDT-positive yet false positives by PCR. Pooled PCR led to the identification of one *P. falciparum* and one *P. malariae* infection among RDT-negative participants. The *P. falciparum*-infected participant reported recent travel to Mozambique. Compared to performing individual testing on thousands of samples, PCR pooling reduced labor and consumable costs by 95.5%. Seropositivity was associated with age ≥20 years (11·7% *vs* 1·9%, *P*<0.001), recent travel to Mozambique (OR 4.4 [95% CI 1.0–19.0]) and residence in southeast Swaziland (RR 3.78, *P*<0.001).

**Conclusions:**

The prevalence of malaria infection and recent exposure in Swaziland are extremely low, suggesting elimination is feasible. Future efforts should address imported malaria and target remaining foci of transmission. Pooled PCR and ELISA are valuable surveillance tools for guiding elimination efforts.

## Introduction

Global progress in malaria control has led to increased interest in and optimism for elimination. Of 99 countries that remain endemic, 32 are moving towards elimination, including four in sub-Saharan Africa [1]. To guide planning, improved diagnostic and surveillance strategies specific to low endemic settings are needed to track progress and identify residual foci of transmission [2], [3].

The burden of malaria is typically estimated by passive and active surveillance. Passive surveillance generally involves health facility based reporting of malaria cases, which can be limited by incomplete reporting, healthcare seeking in the private sector, and poor diagnostic capacity, particularly in low transmission settings where health workers see few malaria cases. Active surveillance addresses some of these limitations and generally involves cross-sectional surveys of defined sample populations, where the primary malaria indicator is the proportion of persons infected with malaria parasites (parasite prevalence) [4]. These surveys enable detection of asymptomatic infections that perpetuate transmission [5] and provide an opportunity to concurrently assess coverage of malaria interventions [6].

Estimating parasite prevalence typically relies on microscopy, which is time and labor intensive, and often inaccurate in operational settings [7], [8]. Newly available rapid diagnostic tests (RDTs) offer ease of use and timely results, but have limitations in specificity, sensitivity, quality, and cost [8]. PCR provides enhanced sensitivity but results are not available immediately. Additionally, when prevalence is low, high costs and labor may render individual testing of every sample impractical. However, a novel pooled method offers the opportunity for efficient, sensitive, and specific testing [9]. Lastly, serology to detect antimalarial antibodies, as a unique measurement of past infection, may help to delineate temporal and spatial trends in transmission [10], [11]. Moreover, in previous elimination programs, absence of antibody responses in certain age groups has been used as evidence of the cessation of transmission [12].

Swaziland is a country in southern Africa that has experienced recent declines in malaria burden [13]. From 1999 to 2010, annual laboratory-confirmed malaria cases decreased from 4005 to 196 (3.8 to 0.2 per 1000 population), and suspected or non-laboratory confirmed cases decreased from 29,374 to 3470 (27.7 to 2.9 per 1000 population) [14], [15]. These declines have been attributed to annual indoor residual spraying (IRS) and a cross-border program that has successfully decreased malaria transmission in neighboring southern Mozambique and South Africa [13]. In 2007, the Ministry of Health with support from regional leaders [16], [17] launched a goal to eliminate malaria by 2015 [18]. To evaluate the impact of the current program, and guide planning for malaria elimination, a national Malaria Indicator Survey (MIS) [6] was conducted for the first time in Swaziland's history. Intervention coverage was assessed and parasitemia was measured by RDT instead of microscopy. Novel surveillance techniques using dried blood spots included pooled PCR, to more accurately estimate parasite prevalence, and serology to estimate past exposure to parasites.

## Results

### Survey demographics and intervention coverage

5613 participants living in 1751 households were surveyed. Most participants lived in urban areas (53.3%), and in the eastern region of Lubombo (79.2%). Twelve percent of participants reported international travel in 2010, with South Africa (84.3%) and Mozambique (14.4%) being the most frequent destinations. Most participants resided in their current residence for >3 years (76.1%) (Table 1).

**Table 1:** Baseline characteristics of survey participants.

Baseline characteristic	No. (weighted %, 95% CI)
Male (n = 5613)	2721 (48.6%, 47.0–50.2)
Age category (n = 5611)	
<10 years	1463 (24.7%, 23.3–26.1)
10–19 years	1221 (21.0%, 19.3–22.7)
≥20 years	2929 (54.3%, 52.4–56.2)
Urban residence (n = 5613)	2186 (53.3%, 48.7–57.9)
Mean altitude, meters (n = 5557)	345.9 (325.4–366.4)
Region (n = 5613)	
Hhohho	782 (10.2%, 5.0–15.3)
Lubombo	4054 (79.2%, 72.8–85.6)
Manzini	334 (4.2%, 1.0–7.3)
Shiselweni	443 (6.5%, 2.5–10.6)
Residence in current district (n = 5199)	
<1 year	528 (11.2%, 9.7–12.7)
1–3 years	619 (12.7%, 11.4–14.1)
>3 years	4052 (76.1%, 74.0–78.1)
International travel in 2010 (n = 5199)	559 (11.6%, 9.9–13.3)

Indoor residual spraying (IRS) in the past 12 months was reported in 44.5% of households. Bed net use was low: 3.6% of participants reported sleeping under an ITN the previous night. Among children and women who sought care for fever in the last two weeks, 0% and 15.4%, respectively, received a finger or heel stick for malaria diagnosis (Table 2). Among 46 participants (0.9%) that reported receiving an antimalarial in the previous 2 weeks, none reported receiving an artemsinin-based combination therapy, the recommended first-line treatment.

**Table 2:** Intervention coverage among of survey participants.

Intervention	No. (weighted %, 95% CI)
Vector control	
Household sprayed in the past 12 months (n = 1751)	773 (44.5%, 40.3–48.6)
Household with at least 1 insecticide treated bed net (ITN) (n = 1751)	301 (16.6%, 14.1–19.1)
Household with at least 1 ITN and/or sprayed in last 12 months (n = 1751)	926 (53.2%, 49.4–57.0)
Slept under an ITN the previous night, all participants (n = 5613)	176 (3.6%, 2.5–4.6)
Slept under an ITN the previous night, children <5 years (n = 766)	41 (5.8%, 3.3–8.4)
Slept under an ITN the previous night, women (n = 1296)	49 (3.8%, 2.5–5.2)
Diagnostic/ treatment service	
Diagnosed with malaria within the past 2 weeks (all participants, n = 5411)	45 (0.9%, 0.4–1.3)
Treated with an antimalarial drug in the past 2 weeks (all participants, n = 5411)	46 (0.9%, 0.5–1.4)
Children with fever in the last 2 weeks and sought care (n = 38)*	26 (67.4%, 51.6–83.1)
Children with fever in the last 2 weeks and received finger or heel stick (n = 38)*	0
Women with fever in the last 2 weeks and sought care (n = 45)**	22 (53.5%, 40.7–66.3)
Women with fever in the last 2 weeks and received a finger stick (n = 22)**	3 (15.4%, 0–33.9)

*Total number of children with fever in last 2 weeks (n = 362): 39 (12.5%, 95% CI 6.7–18.3%).

**Total number of women with fever in last 2 weeks (n = 961): 47 (4.7%, 95% CI 2.6–6.8%).

### RDT and PCR results

**Figure 1:**
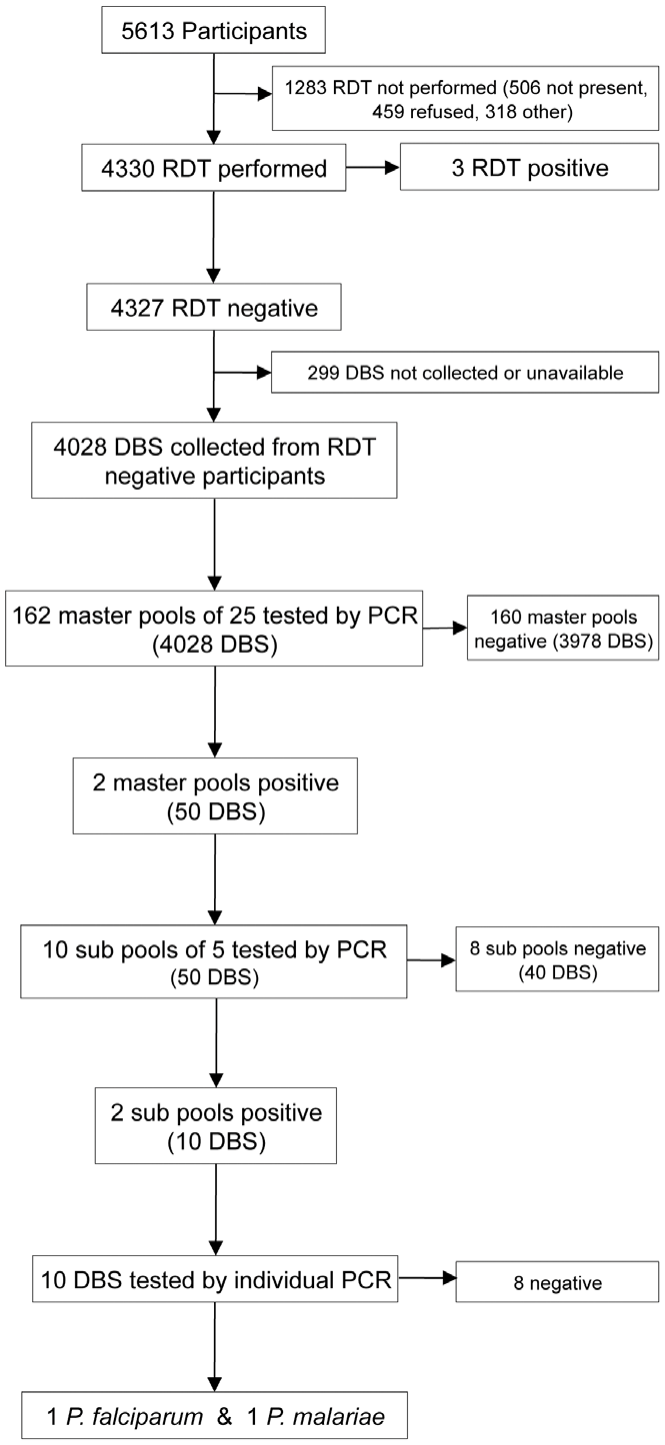
Participant recruitment and results for testing by RDT and pooled-PCR. DBS, dried blood spots.

**Figure 2:**
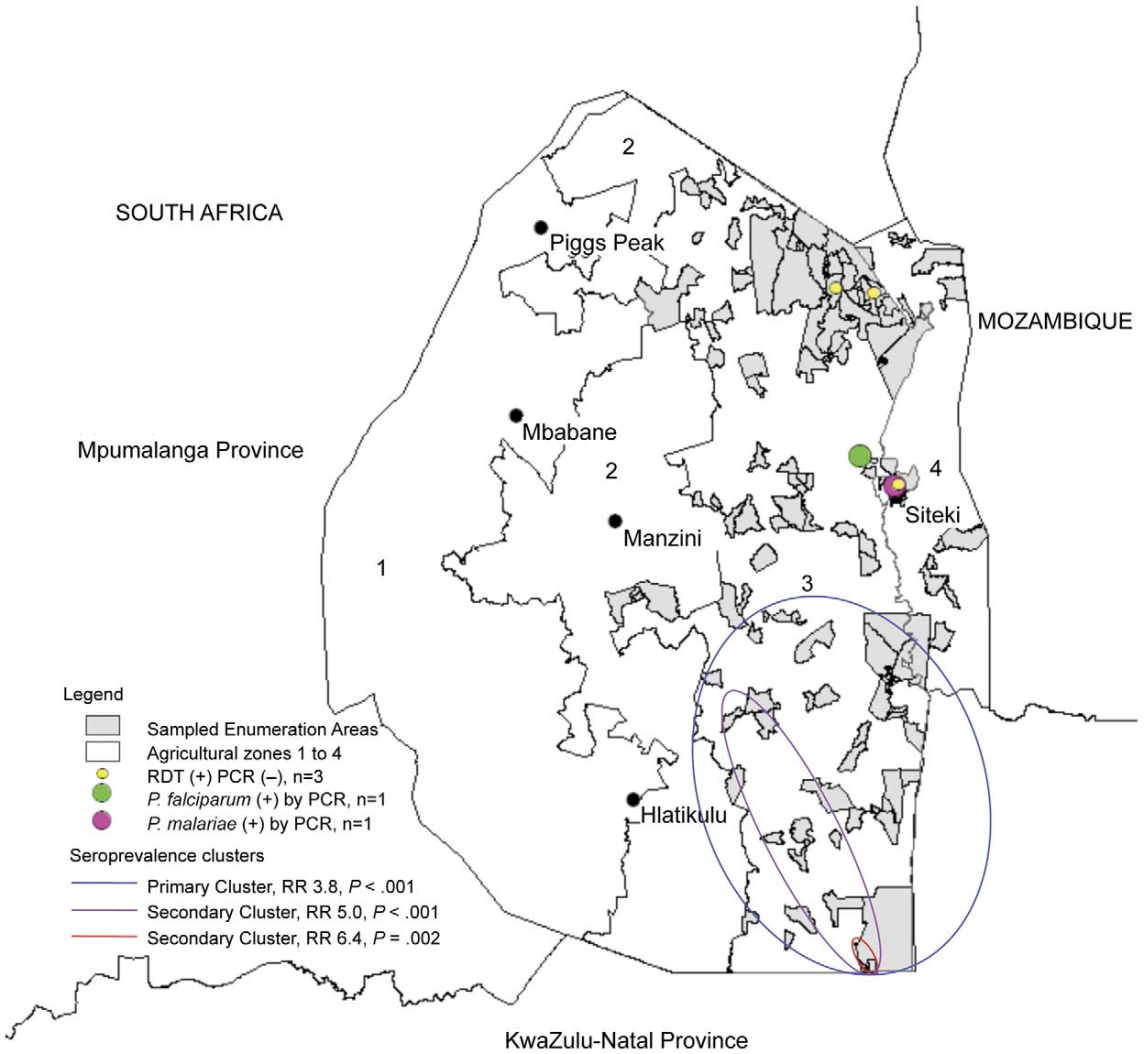
Map of Swaziland with RDT or PCR-positive participants and potential hot spots identified in serologic cluster analysis.

RDTs were performed in 4330 of 5613 (77%) participants and three were positive for *P. falciparum* (Figure 1). By PCR, these were negative in duplicate and therefore considered false positives. 4028 dried blood spots from RDT-negative participants were tested by PCR using the three-stage pooling strategy. Two infections were identified, one *P. falciparum* in a 57 year-old man, and one *P. malariae* in a 46 year-old woman. Both resided in the northeast region of Swaziland, near the Mozambique border (Figure 2). Neither reported having a fever in the two last weeks, though the *P. falciparum*-infected participant reported recent travel to Maputo, Mozambique. Based on PCR results, weighted parasite prevalence was estimated at 0.2% (95% CI 0.0–0.6). Using PCR as the gold standard, RDTs identified none of the two PCR positive samples resulting in a sensitivity of 0.0%, specificity of 99.9%, positive predictive value of 0%, and negative predictive value of 100%.

### Cost and operational comparisons between different methods used to identify infection

Using a pooled strategy for PCR, a total of 182 assays were performed to test the 4028 dried blood spots (Figure 1). Compared to performing individual PCR on all samples, use of pooling reduced labor and consumable costs by 95.5%.

Cost and other operational issues were considered in a comparison amongst RDT, microscopy, individual PCR, and pooled PCR (Table 3). Cost to assay all samples was most expensive by individual PCR and RDT (>$6000 USD). Pooled PCR had the highest facility, equipment, and training needs, but testing costs were the lowest ($291). Only RDT could be used as a point of care test and thus guide treatment during the survey. Turnaround time was on the order of weeks for microscopy and individual PCR, and on the order of days for pooled PCR.

**Table 3:** Cost and operational comparisons between infection diagnostic methods.

Cost or Operational Issue	RDT	Microscopy	PCR
Cost per sample*	$1.50	$0.26	$1.55
Cost for all 4031 samples*	$6047	$1048	$6248 (individual PCR)$291 (pooled PCR)
Detection limit**	100 to 200 p/µL	4 to 100 p/µL	<4 p/µL (individual PCR) 100 p/µL (pooled PCR)
Point-of-care test	Yes	Only if basic laboratory services available	Not practical
Capital equipment	None	Microscope	PCR machine
Training	Minimal	Moderate	Extensive
Turnaround time per sample	15 minutes	30 minutes	2 days
Turnaround time for all 4031 samples	n/a	Weeks	Weeks (individual PCR)Days (pooled PCR)

p/µL = parasites/µL.

*Costs presented in US$ and do not include personnel. Estimates for microscopy were approximated based on published figures [8], and estimates for RDT and PCR were gathered from this study.

**Estimates based on published figures [8], [9], [22]. Detection limit for pooled PCR based on use of a 100 parasites/µL sample as control in this study. However, it should be noted that detection limit <4 parasite/µL for pooled PCR is possible as previously reported [9].

### Serology results

**Figure 3:**
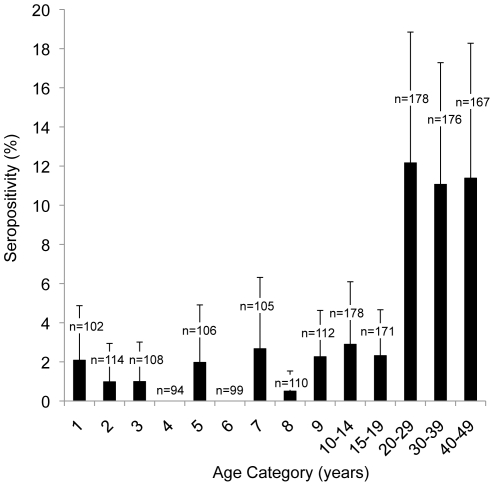
Seroprevalence to *P. falciparum* antigens MSP-1_42_ and AMA-1 by age category with 95% confidence interval half-widths.

1820 of 4031 participants with dried blood spots were sampled for serology. Overall, 82 participants (5.8%, 95% CI 3.9–7.6) had antibodies to both AMA-1 and MSP-1_42_ antigens. There was a step increase in seroprevalence at 20 years of age (Figure 3). Compared to participants that were 20 to 49 years of age, seroprevalence among participants one to 20 years of age was significantly lower (1.9% vs 11.7%, *P*<0.001). Exclusion of participants who reported recent travel to Mozambique yielded the same age-specific pattern of seroprevalence (data not shown). Analysis of seroconversion rates suggested a significant change in seroconversion rate 20.1 years ago (95% CI 18.8−20.5), suggesting a temporal change in malaria exposure in the year 1989.

There were no significant relationships between seropositivity and wealth index, urban vs rural residence, altitude, IRS coverage, or bed net use. Reported travel to Mozambique, but not South Africa, within 2010 was significantly associated with seropositivity (OR 4.4, 95% CI, 1.0–19.0, *P* = 0.048).

Spatial cluster analysis was performed to identify areas with higher than expected seroprevalence. A primary cluster was identified in the southeast region of the country (RR for seropositivity 3.8, *P*<0.001). Two secondary clusters of higher relative risk were identified within the primary cluster: one extending from Sithobela to Somntongo constituency (RR 5.0, *P*<0.001) and a smaller one limited to Somntongo (RR 6.4, *P* = 0.002) (Figure 2). To assess whether clusters of seropositivity reflected only distant exposure, the same analyses were performed among participants less than ten years of age. Similar clusters were identified: the primary cluster extended from Sithobela to Somntongo (RR 5.2, *P* = 0.02) and a secondary cluster was located in Somntongo (RR 6.9, *P* = 0.03). Analyses examining responses to individual antigens or either antigen showed similar clustering (data not shown). Analysis excluding participants with reported recent travel to Mozambique yielded the same three clusters.

## Discussion

To guide strategic planning for the transition from malaria control to elimination, we utilized novel malaria surveillance techniques of pooled PCR and serology to perform the first large scale cross-sectional malaria survey in Swaziland and found that prevalence of infection and recent exposure was extremely low. By pooled PCR, only one *P. falciparum* and one *P. malariae* infection were identified among 4028 participants. Compared to RDT, pooled PCR identified infections missed by RDT and provided improved efficiency and affordability excluding capital costs. Serological data identified a potential focus of recent transmission in the southeast and low seroprevalence in younger age groups, suggesting low exposure to malaria in recent years. Report of recent travel to Mozambique was identified as a risk factor for both *P. falciparum* infection and seropositivity.

Large-scale prevalence surveys have traditionally relied on microscopy, but considering the significant time and labor required, and potential operational limitations, the Swaziland malaria program decided to use RDTs. As a simple point-of-care test, RDTs are convenient, but can give false positive results with underlying autoimmune conditions, non-malarial infections, or persistence of the *P. falciparum* HRP-2 antigen despite resolution of infection [19]. Pooled PCR is extremely specific, 100%, because repeat testing of the sample at each stage limits DNA contamination [9]. Using pooled PCR as gold standard, there were three false positives by RDT in our study, and due to the extremely low prevalence, positive predictive value was poor. With large-scale surveys in higher prevalence settings, RDT have also had low positive predictive value [20].

RDTs may also miss infections of low parasite density and many do not detect non-falciparum species [8]. In our study, the *P. falciparum*-specific RDT missed one *P. malariae* infection, and one *P. falciparum* infection that was likely of low parasite density given that the participant was afebrile. Others have found low parasite density to be a determinant of decreased sensitivity in survey settings [21]. The detection limit of current RDTs is 100–200 parasites/µL. The detection limit of pooled PCR can reach submicroscopic levels but sensitivity is only reliable at 100 parasites/µL [9]. It is possible that the three RDT positives were true infections missed by pooled PCR. As an antigen-based assay, RDT could potentially be more sensitive if there is sequestering of *P. falciparum* parasites, particularly in low density infections. These subjects could have also had a recent infection that cleared, but persistent antigenemia. While not assessed in this study, microscopy and individual PCR are more sensitive than pooled PCR [9]. However, the efficiency provided by pooled PCR, and potential for improved sensitivity over RDT, suggest that for large-scale surveys among asymptomatic populations in low prevalence settings, pooled PCR may be preferred. Compared to performing individual PCR testing, use of pooling in this study reduced labor and consumable costs for PCR by greater than 95%.

One limitation with measuring parasitemia in a cross-sectional survey is that regardless of the sensitivity of the test, only one moment in time is captured. In low endemic settings such as Swaziland, prevalence may be so low that it will be difficult to track progress toward elimination. As a measure of past infection, serology has been proposed as a useful way to estimate exposure in low endemic settings [2], [10], [22]. In our study, a statistically significant difference in seroprevalence and seroconversion rate among participants less than 20 years of age compared to older participants suggests a significant decrease in transmission in 1989, with low stable levels of transmission since that time [23]. Declining incidence in Swaziland over the past 20 years is consistent with this finding [14], [15], [24].

Serological data may additionally help to identify other epidemiological risk factors for malaria. Travel to Mozambique and residence in the southeastern region were found to be associated with seropositivity. The first finding is consistent with Swaziland's passive surveillance and case investigation data, which have found travel to Mozambique to be a risk factor for infection [25]. The second finding was unexpected as incidence in the southeastern region of the country has been low in recent years [25]. However, there are many sugar plantations in this area and migrant workers from Mozambique may be a source of transmission. Our data did not include information on occupation or nationality, but suggest that detailed, focal investigation of this region is indicated to determine if seropositivity represents past infection acquired in Mozambique or local transmission.

One potential limitation in our study is that cutoff for seropositivity was based on an assumption of a bi-modal distribution of seropositives and seronegatives within the population sampled. Samples from representative seronegatives (e.g. age- and genetically-matched participants from Swaziland with no history of falciparum exposure but similar exposure to other infections that could result in cross-reactive antibodies) were not available and in practice may be difficult to obtain. Our data suggest a clear temporal trend of a decline in malaria transmission 20 years prior. However, the potential non-specific response seen among younger age groups may suggest that this method is not specific enough to show cessation of transmission, as has been shown in other settings [12]. To enable more accurate estimates of further decrease in transmission using serology, longitudinal studies are needed to better characterize the development, maintenance, and decay of specific antibodies [22].

Findings of this study have important implications on Swaziland's strategic planning for malaria elimination. First, the findings of extremely low parasite prevalence and recent exposure suggest that the high IRS coverage (45% compared to other parts of Africa, where IRS coverage is approximately 10% [15]) and the cross-border collaboration with Mozambique and South Africa have been effective and should be continued. Second, low transmission in spite of low ITN use suggests that further investment into this costly program may not be justified. Rather, ITNs could be reserved for use in identified hot spots and high-risk groups. Third, limited access to diagnostic and treatment services among febrile participants identified passive surveillance and case management as an area for improvement. Finally, future efforts should aim to prevent imported malaria and investigate and target interventions to limited foci of transmission.

For low transmission countries aiming to eliminate malaria, reliance on microscopy or RDT for active surveillance may be inadequate. We document the first national survey from an elimination setting to show that pooled PCR and serology, as accurate and efficient methods to measure current and past infection, can provide critical data to inform strategic planning. Additionally, renewed interest in elimination has been accompanied by skepticism about the feasibility of elimination, particularly in sub-Saharan Africa [26]. This study documents an extremely low prevalence of malaria infection and recent exposure in Swaziland, providing evidence-based optimism for efforts in Swaziland and the region.

## Materials and Methods

### Objectives

We aimed to measure and describe the burden of malaria in Swaziland as relevant to planning for malaria elimination. We hypothesized that compared to using RDT, use of pooled-PCR and serology would improve sensitivity and efficiency for detection of infections and use of serology would clarify temporal and spatial trends in exposure.

### Participants

The historically malaria endemic eastern part of the country was included. Based on the ecological and administrative subdivisions of the country, census enumeration areas (EAs) were arranged into four strata. Of 598 EAs, 172 were randomly selected based on probabilities proportional to population size. Geo-coordinates of all households within selected EAs were recorded [27], [28]. Initially, 15 households were randomly selected within each EA; due to time and budget constraints, ten households were targeted as the study progressed.

### Procedures

An MIS with a stratified two-stage cluster sample, cross-sectional design was used to generate representative estimates of intervention coverage and malaria disease burden. Given Swaziland's goal of elimination, several modifications were made to the traditional MIS design [6]. Intervention coverage and parasite prevalence were assessed in all age groups, not just children less than five years of age and women of reproductive age. Additional questions about travel in 2010 and residence were included, and microscopy and hemoglobin testing were excluded in favor of RDT, pooled PCR, and serology. Data and sample collection took place April to May of 2010, near the end of the annual high transmission season. A household questionnaire was administered to the household head or other consenting adult. A women's questionnaire was administered to women 15 to 49 years of age.

### Parasite Detection

A single finger prick was performed to collect blood for RDT (First Response Malaria Ag *P. falciparum* HRP2 Detection Rapid Card Test, Premier Medical Corporation Ltd) and Whatman 903 filter paper. Participants testing positive by RDT were transported to the nearest health facility for care.

Blood spots were dried for at least three hours and stored in individual plastic bags with desiccant at ambient temperature. They were transferred to 4°C within one week and to −20°C within one month. In duplicate, dried blood spots from RDT-positive participants were individually chelex extracted then tested by PCR using nested PCR targeting the *cytochrome b* gene [9].

### Pooled PCR Methods

Samples from RDT-negative participants were tested using a three-stage PCR-based pooling strategy as previously described [9], [29]. Briefly, samples were first tested in master pools of 25. Positive master pools were divided and tested into sub pools of five, with positive sub pools tested as individual samples. At each stage, DNA was chelex extracted from pooled or individual dried blood spots, and then tested using nested PCR targeting the *cytochrome b* gene. In pooled stages, controls reflected the test pool size, e.g. controls for the master pool stage consisted of one punch from a laboratory generated dried blood spot (parasite density 100 parasites/µL) mixed with 24 negative spots. To determine species, PCR-positive samples underwent an *AluI* restriction digestion and were compared to digestion patterns of known controls [30].

### Serology sampling and testing

A sub-sample of all participants with a dried blood spot was selected for serologic testing. Those less than one year of age were excluded due to potential persistence of maternal antibodies. All participants one to nine years of age were included because results in this age group were expected to be most reflective of recent changes in transmission. Based on reported declines in incidence, age-stratified analyses were expected to be powered by a lower seroprevalence in younger age groups. Therefore, participants ten to 49 years of age were randomly sampled by district based on the maximum number of participants in the younger age categories. Participants 50 years of age and older were excluded.

### ELISA Methods

ELISA assays were performed similarly to previously described methods [31]. A 3 mm punch was excised from the dried blood spot and antibodies were eluted overnight in 240 µL phosphate buffered saline with 0.05% Tween 20 (PBS-T), which was also used for all washing steps. Elutes were assayed in duplicate for antibodies against *Plasmodium falciparum* FVO strain blood stage antigens merozoite surface protein-1 (MSP-1_42_), and apical membrane antigen-1 (AMA-1), provided by Walter Reed Army Institute of Research [32]. High absorbance plates (Immulon 4HX) were coated with 75 µL of antigen at 0.5 µg/mL overnight. Plates were washed and then blocked using 150 µL of 5% Blotto, non-fat milk in phosphate buffered saline. After washing, plates were incubated with 75 µL of elutes as well as titrations of pooled serum from previously infected participants in Kampala, Uganda. After another washing step using PBS-T, plates were incubated with 75 µL of antihuman IgG antibody (AP-conjugated AffiniPure Goat Anti-Human IgG, Jackson Immunoresearch). After a final washing step, 75 µL of substrate (BluePhos Microwell Phosphatase Substrate System, KPL) was added and optical density detected using Versamax ELISA reader (Molecular Diagnostics).

### Data management and statistical analyses

The sample size was generated to provide an estimate of insecticide-treated bed net (ITN) use in children less than five years of age. Based on previous surveys, response rate and design effect were estimated at 90% and 1.3, respectively, allowing for a sampling error of 12% [33]. With the average number of children under five per household being 0.13, 2500 households were targeted.

Survey data were entered into personal digital assistants (PDAs) and downloaded into Microsoft Access (2007). Analyses were performed using SAS (version 9.2) and STATA (version 11.0). Point estimates and confidence intervals were calculated incorporating survey procedures and weights to adjust for multi-stage clustering and changing selection probability.

For serologic analyses, raw optical densities were standardized by dividing values by a positive control on all plates. Samples with a coefficient of variation >0.3 between duplicates were repeated. To determine seropositivity cutoffs for each antigen, standardized optical densities were fitted to a mixture model that assumed a bi-modal normal distribution, and seropositivity was defined as three standard deviations above the mean of the lower distribution [31]. Due to the potential for false positive responses to a single antigen, seropositivity was defined as presence of antibodies to both AMA-1 and MSP-1_42_.

Evidence for temporal changes in exposure was explored by assessing the relationship between age and seroprevalence. To formally assess a temporal change in exposure, a catalytic conversion model assessing seroconversion rate was fitted to the data, allowing for a change in the seroconversion rate at a single time-point. The time point at which a change in seroconversion occurred was assessed by maximum likelihood with confidence intervals based on the chi-squared distribution on one degree of freedom [23].

To analyze relationships between seropositivity and baseline characteristics, chi-squared, t-test, logistic regression, or two-sample test of proportions was performed as appropriate. *P*-values less than .05 were considered statistically significant. To identify potential foci of transmission, spatial cluster analysis was performed with SatScan (version 9.0), using the Poisson model and allowing for elliptical clusters. Due to sampling among participants ten to 49 years of age, age was adjusted for as a categorical variable (<10 years, 10-19 years, ≥20 years). Maximum geographical cluster size was set at 50% of the population, allowing for nested clusters. Statistically significant clusters were reported, including nested clusters with a relative risk greater than that of the parent cluster. Maps were produced using ArcGIS software (version 10.0).

### Ethics

For the household and women's questionnaires, oral informed consent was provided by a household head and women 15 to 49 years of age, respectively. Written consent was not performed because data along with oral consent was entered electronically into PDAs, and there were no sensitive questions and thus minimal risks for participants. Written informed consent for blood testing was obtained from participants or a parent or guardian for children less than 14 years of age. Ethical approval for the study, including the use of oral consent for the questionnaires, was obtained from the review committees at the Swaziland Ministry of Health, University of California, San Francisco, and United States Centers for Disease Control and Prevention.
